# Effect of Nursing Workload in Intensive Care Units

**DOI:** 10.7759/cureus.12674

**Published:** 2021-01-13

**Authors:** Ameenah A Almenyan, Asma Albuduh, Fatimah Al-Abbas

**Affiliations:** 1 Nursing, Ministry of Health, Al Ahsa, SAU; 2 Medicine, Ministry of Health, Al Ahsa, SAU

**Keywords:** icu, nursing, workload, adverse events, complications

## Abstract

Nursing work efforts are important in providing sound healthcare services, especially in the intensive care units (ICU). Complications and adverse events are more liable to occur among patients in the ICU and these patients require more attention and nursing care. Most of the research in this field is mainly focused on the effect of staffing and its correlation to patient safety and satisfaction. Previous studies also showed that reduced nursing staffing was significantly associated with the development of pneumonia in ICU patients who needed more nursing requirements. An increase in nursing workload is also significantly associated with an increased incidence rate of nosocomial infections. The association between nursing workload in ICU patients and increased incidence rates of mortality is also supported by previous studies. The nurse-to-patient ratio has been previously used to evaluate patient safety correlation with the nursing workload as reported by previous reports. However, previous research shows that the nursing workload is a more complex correlation and can not be determined by a simple ratio as the nurse-to-patient one. Evidence shows that many adverse events may occur with patients in the ICU secondary to reduced nursing care such as increased mortality, length of hospital stay, and catching in-hospital infections. In the current study, we aim to review the outcomes from previous investigations to further emphasize the effect of nursing workload on ICU patient outcomes and safety.

## Introduction and background

The recently increasing demand in healthcare resources is influenced by many factors such as aging population, epidemiological changes, technological advances, and emerging epidemics. This may have led to many changes being applied recently in the healthcare management system leading to a better quality of care and decreased costs [1]. Nursing activities are important in providing sound healthcare services, especially in the intensive care units (ICU). However, such activities are variable based on many factors like working atmosphere, disease severity, workload, personnel qualifications and skills, and cost-efficacy together with the determined clinical outcomes of the diseased patients [2]. Providing adequate nursing staffing has become an essential key-element in maintaining and providing better care quality that results in better patient satisfaction and improved clinical outcomes.

A study by Aiken et al. [3] showed that an increased nurse-to-patient ratio is significantly associated with reduced mortality rates. On the other hand, a reduced nurse-to-patient ratio by increasing the number of patients or reducing the number of nurses is correlated with increased workload. As a result, it can lead to job dissatisfaction, reduced efficiency, and increased complications and mortality rates among patients [4-6]. Therefore, it is essential to reconsider healthcare resource distribution and nursing staffing based on the workload of the affected centers to prevent any complications such as nosocomial infections​​​​​ [7-9]. These complications and adverse events are more liable to occur among patients in the ICU as these patients require more attention and nursing care. Aiken et al. [3] estimated that around 20% of patients in the ICU usually experience additional adverse events with low nurse-to-patient ratio.

Evidence from the current literature shows that many assessment approaches should be conducted for better care of patients in the ICU. These include pneumonia and nosocomial infections, bed ulcers, fractures, possible adverse events from potential medications, and others [10]. These can be used to increase the quality of the provided care in achieving a patient efficient integrated nursing crew. Consequently, it is essential to maintain good nursing habits for patients in the ICU which requires the integration of managerial, and organizational assessment of such activities to provide improved healthcare. As the previous study showed, nursing workload is significantly associated with ICU patients outcomes and safety. In the current review, we aim to discuss the outcomes from previous investigations to further emphasize the effect of nursing workload on ICU patient outcomes and safety.

## Review

Effect of nursing workload on ICU patients

An increase in nursing workload results in a reduced patient survival rate which in return may be attributable to the increased suboptimal care for some patients [11]. As a result, it may affect the overall required care for some of the patients [11,12]. The Agency for Healthcare Research and Quality (AHRQ) studied the correlation between hospital nursing workload and staffing and its effect on patient outcomes and safety [12]. They concluded that most of the research in this field mainly focused on the effect of staffing and its correlation to patient safety and satisfaction. Evidence from previous investigations showed that reduced staffing is significantly associated with many nursing-sensitive outcomes related to nursed patients [13,14]. Furthermore, previous studies that were applied on existing databases of more than 124 thousand patients also concluded that a reduced nursing staffing was significantly associated with the development of pneumonia in patients who required more nursing care [15-17]. A previous multicentre study in the United States showed that patients admitted for surgeries had a higher risk in developing pneumonia and was significantly reduced by 8.9% when nursing staffing was increased by one hour per day for these patients. so, an increased time of care can be achieved by increased staffing [15]. Another study from the United States also showed that lower pneumonia rates were significantly correlated with increased nursing staffing in the included hospitals [18,19]. Therefore, it should be noted that staffing is not the only significant factor that can contribute to the overall effects of reduced nursing care and the workload of nurses should be further emphasized [18,19]. Factors such as economy, pandemics, size of center, and patient load can play more role and should be explored in future research.

Evidence showed that an increased rate of nursing workload was significantly associated with increased rates of nosocomial infections. Among surgical patients, a higher proportion of care provided by registered nurses was associated with lower rates of urinary tract infections (P=0.04), and a greater number of hours of care per day provided by registered nurses was associated with lower rates of “failure to rescue” (P=0.008) [14]. Moreover, Harbarth et al. conducted a study in the neonatal ICU to investigate the same correlation and found that reduced nursing staffing was significantly associated with increased incidence rates of E cloacae infections [20]. Similarly, a study by Archibald et al. in Philadelphia in Philadelphia, United States showed that the monthly incidence rates of nosocomial infections were significantly lower with the reduced working hours of the registered nurses [21].

Needleman et al. [14] studied 11 centers across the United States and stated that lower incidence rates of failure to resuscitate as well as high mortality rates were associated with the increased nursing hours provided by the registered healthcare workers [14]. The increased workload per each patient showed that it can contribute to overall in-hospital mortality. Aiken et al. concluded that if the workload of a registered nurse was increased by one patient, this will contribute to a 7% increase in the risk of mortality in the corresponding population [11]. Manheim et al. also reported that increasing the hospital staffing nurses can reduce the mortality rates and increase the efficiency of the introduced care [22]. A previous investigation by Pronovost et al. in 1999 showed that, in patients undergoing abdominal aortic surgeries, the rate of hospital stay for these patients increased by 20% when the ratio for nurses to patients was less than 1:2 per day [23]. Lichtig et al. also proved that increased nursing workloads, represented by increasing work hours, were significantly associated with reduced complications and hospital stay [24]. In Australia, a study by Beckmann et al. showed that reduced nurses to patients ratio in the ICU was significantly associated with increased rates of drug self-administration, ventilation, self-extubation, lack of supervision and documentation [25]. Moreover, the need to be mechanically-ventilated secondary to acquired pneumonia was significantly associated with reduced care hours provided by the registered nurses [2].

A previous meta-analysis of nine observational studies concluded that reduced mortality rates in the ICU were not significantly associated with increasing the level of nursing workload and staffing. However, the same study showed that their results were not conclusive due to flawed errors in the included studies [26]. Thompson et al. conducted a large observational study in 35 hospitals and 45 ICUs and reported a correlation between increased nursing working hours and quality of provided care [27]. The authors reported that increased working hours by 20 hours per week significantly increases the risk of catching a nosocomial infection and increases the length of hospital stay for the corresponding patients. In another study by Chang et al., the nurse-to-patient ratio was significantly associated with in-hospital mortality rates, patients’ resuscitation, and overall quality of the provided care [28]. Jung et al. also conducted an observational study to find the correlation between in-hospital mortality and nursing workload among the included population [29]. They divided their population into four grades based on the estimated bed-to-nurse ratio of the center. The authors reported that in-hospital mortality rates were significantly associated with an increased bed-to-nurse ratio (<0.63 or more). However, significance was not estimated in other patients that did not require mechanical ventilation. This was justified by the fact that mechanical ventilation requires the integration of more efforts by the accompanying nurses (due to additional medications, procedures, and equipment) which add to the overall workload and reduces the efficacy of these nurses [30,31]. The association between nursing workload in ICU patients and increased incidence rates of mortality. For instance, Neuraz et al. in an observational study found that when patient-to-physician ratio exceeded 14 on the Therapeutic Intervention Scoring System (TISS), patients' safety becomes compromised [32,33].

The nurse-to-patient ratio has been previously used to evaluate patient safety correlation with the nursing workload as reported by previous reports. However, previous research shows that the nursing workload is more complex and can not be determined by a simple ratio as the nurse-to-patient one. This nurse-to-patient ratio is taken into account because of its availability and easy-to-use structure [34]. On the other hand, a previous meta-analysis suggested that having an adequate nurse-to-patient ratio might be a significant factor in reducing the adverse events faced by the patients in the ICU in addition to reducing the healthcare costs by preserving the potential resources and reducing the hospital stay [35]. Another meta-analysis by Kane et al. [36] analyzed the results of 28 studies and reported that lower mortality rates among patients in the ICU were significantly associated with increased nursing staffing. However, the study estimated that lower cardiac arrest, unexplained extubation, nosocomial pneumonia, and respiratory failure rates were significantly reduced when the registered number of nurses increased by 1 per patient.

Nursing workload and patient safety

Reports by the Systems Engineering Initiative for Patient Safety (SEIPS) indicated that nursing workload can directly affect the quality of the care provided for the patients which in return affects their safety [37]. Lack of time and increased tasks for nurses in the ICU, which is attributable to the increased workload, is a significant reason for such an effect. Nurses may face huge difficulties in integrating the required tasks and providing the needed care for patients in need. Griffith et al. expressed that increased consumption of healthcare resources may impact the ability of the healthcare officials to direct nursing capabilities into the right path [38]. Baggs et al. also indicated that increased workload may affect the quality of the provided care as nurses would have less time to spend with the relative physicians which increases the risk for developing mistakes and decreasing the quality of care [39]. Reduced communication intervals between the nurses and patients might also be a contributing factor. Cavanagh et al. mentioned that increased workload might lead to job dissatisfaction which leads to poor performance affecting the quality of the provided care [40]. This theory has been proved by previous investigations as researchers showed that patient satisfaction will lead to a better quality which may result from high-quality care attributable to the satisfaction of the job [41,42]. In addition to these factors, the increased workload might also lead to a higher burnout and stress levels of the nurses. This may impact the nurses’ abilities to work and reduces the efficiency of the provided care owing to the reduced cognitive and physical capabilities, which can affect the quality of the provided care for the patients [43,44]. Mistakes and errors might also increase with more workload on the performing nursing crew [45]. To eliminate the shortage of nursing efforts attributable to the increased workload, factors that contribute to such events must be identified and corrected. Carayon et al. [46] reported that such factors include the relativity and availability of nurses staffing per patient, exhaustion and stress, poor equipment, and easy access to the patient. Therefore, it is essential to propose human-engineering based approaches suitable for these issues to decrease the burden resulting from the increased workload of nursing personnel [46,47].

## Conclusions

The nurse-to-patient ratio has been previously used to evaluate patient's safety relation with the nursing workload as reported by previous reports. However, previous research showed that the nursing workload is a more complex correlation and can not be determined by a simple ratio as the nurse-to-patient one. This nurse-to-patient ratio is taken into account because of its availability and easy-to-use and probably be used as a baseline for evaluating all the factors involved in ICU care. Evidence shows that many adverse events can occur with patients in the ICU secondary to reduced nursing care such as mortality, increased hospital stay, and catching in-hospital infections. Further investigations should be directed towards the rightful redistribution of the nursing capabilities based on the available healthcare resources to prevent such adverse events.

## References

[1] Rhodes A, Moreno RP (2012). Intensive care provision: a global problem. Rev Bras Ter Intensiva.

[2] Nogueira TdA, Menegueti MG, Perdoná GdSC, Auxiliadora-Martins M, Fugulin FMT, Laus AM (2017). Effect of nursing care hours on the outcomes of intensive care assistance. PLoS One.

[3] Aiken LH, Sloane DM, Bruyneel L (2014). Nurse staffing and education and hospital mortality in nine European countries: a retrospective observational study. Lancet.

[4] Aragon Penoyer D (2010). Nurse staffing and patient outcomes in critical care: a concise review. Crit Care Med.

[5] de Magalhães AM, Dall'Agnol CM, Marck PB (2013). Nursing workload and patient safety--a mixed method study with an ecological restorative approach. Rev Latino-Am Enfermagem.

[6] Schubert M, Clarke SP, Aiken LH, de Geest S (2012). Associations between rationing of nursing care and inpatient mortality in Swiss hospitals. Int J Qual Health Care.

[7] Nogueira Lde S, Ferretti-Rebustini RE, Poveda Vde B (2015). Nursing workload: is it a predictor of healthcare associated infection in intensive care unit? [Article in Portuguese]. Rev Esc Enferm USP.

[8] Hugonnet S, Uçkay I, Pittet D (2007). Staffing level: a determinant of late-onset ventilator-associated pneumonia. Crit Care.

[9] Marcin JP, Rutan E, Rapetti PM, Brown JP, Rahnamayi R, Pretzlaff RK (2005). Nurse staffing and unplanned extubation in the pediatric intensive care unit. Pediatr Crit Care Med.

[10] Claro CM, Krocockz DV, Toffolleto MC, Padilha KG (2011). Adverse events at the intensive care unit: nurses' perception about the culture of no-punishment [Article in Portuguese]. Rev Esc Enferm USP.

[11] Aiken LH, Clarke SP, Sloane DM, Sochalski J, Silber JH (2002). Hospital nurse staffing and patient mortality, nurse burnout, and job dissatisfaction. JAMA.

[12] Anderson FD, Maloney JP, Beard LW (1998). A descriptive, correlational study of patient satisfaction, provider satisfaction, and provider workload at an army medical center. Mil Med.

[13] Hughes RG, Clancy CM (2005). Working conditions that support patient safety. J Nurs Care.

[14] Needleman J, Buerhaus P, Mattke S, Stewart M, Zelevinsky K (2002). Nurse-staffing levels and the quality of care in hospitals. N Engl J Med.

[15] Cho SH, Ketefian S, Barkauskas VH, Smith DG (2003). The effects of nurse staffing on adverse events, morbidity, mortality, and medical costs. Nurs Res.

[16] Kovner C, Jones C, Zhan C, Gergen PJ, Basu J (2002). Nurse staffing and postsurgical adverse events: an analysis of administrative data from a sample of U.S. hospitals, 1990-1996. Health Serv Res.

[17] Kovner C, Mezey M, Harrington C (2000). Research priorities for staffing, case mix, and quality of care in U.S. nursing homes. J Nurs Scholarsh.

[18] Kovner C, Gergen PJ (1998). Nurse staffing levels and adverse events following surgery in U.S. hospitals. J Nurs Scholarsh.

[19] Unruh L (2003). Licensed nurse staffing and adverse events in hospitals. Med Care.

[20] Harbarth S, Sudre P, Dharan S, Cadenas M, Pittet D (1999). Outbreak of Enterobacter cloacae related to understaffing, overcrowding, and poor hygiene practices. Infect Control Hosp Epidemiol.

[21] Archibald LK, Manning ML, Bell LM, Banerjee S, William JR (1997). Patient density, nurse-to-patient ratio and nosocomial infection risk in a pediatric cardiac intensive care unit. Pediatr Infect Dis J.

[22] Manheim LM, Feinglass J, Shortell SM, Hughes EF (1992). Regional variation in Medicare hospital mortality. Inquiry.

[23] Pronovost PJ, Jenckes MW, Dorman T (1999). Organizational characteristics of intensive care units related to outcomes of abdominal aortic surgery. JAMA.

[24] Lichtig LK, Knauf RA, Milholland DK (1999). Some impacts of nursing on acute care hospital outcomes. J Nurs Adm.

[25] Beckmann U, Baldwin I, Durie M, Morrison A, Shaw L (1998). Problems associated with nursing staff shortage: an analysis of the first 3600 incident reports submitted to the Australian Incident Monitoring Study (AIMS-ICU). Anaesth Intensive Care.

[26] Numata Y, Schulzer M, Van Der Wal R, Globerman J, Semeniuk P, Balka E, FitzGerald JM (2006). Nurse staffing levels and hospital mortality in critical care settings: literature review and meta-analysis. J Adv Nurs.

[27] Thompson D, Hsu YJ, Chang BH, Marsteller JA (2013). Impact of nursing staffing on patient outcomes in intensive care unit. J Nurs Car.

[28] Chang LY, Yu HH, Chao YC (2019). The relationship between nursing workload, quality of care, and nursing payment in intensive care units. J Nurs Res.

[29] Jung M, Park H, Kang D (2020). The effect of bed-to-nurse ratio on hospital mortality of critically ill children on mechanical ventilation: a nationwide population-based study. Ann Intensive Care.

[30] Miranda DR, Nap R, de Rijk A, Schaufeli W, Iapichino G (2003). Nursing activities score. Crit Care Med.

[31] Reis Miranda D, de Rijk A, Schaufeli W (1996). Simplified Therapeutic Intervention Scoring System: the TISS-28 items--results from a multicenter study. Crit Care Med.

[32] Neuraz A, Guérin C, Payet C (2015). Patient mortality is associated with staff resources and workload in the ICU: a multicenter observational study. Crit Care Med.

[33] Tarnow-Mordi W, Hau C, Warden A, Shearer AJ (2000). Hospital mortality in relation to staff workload: a 4-year study in an adult intensive-care unit. Lancet.

[34] Carayon P, Gürses AP (2005). A human factors engineering conceptual framework of nursing workload and patient safety in intensive care units. Intensive Crit Care Nurs.

[35] Thungjaroenkul P, Cummings GG, Embleton A (2007). The impact of nurse staffing on hospital costs and patient length of stay: a systematic review. Nurs Econ.

[36] Kane RL, Shamliyan TA, Mueller C, Duval S, Wilt TJ (2007). The association of registered nurse staffing levels and patient outcomes: systematic review and meta-analysis. Med Care.

[37] Carayon P, Hundt AS, Alvarado CJ, Springman SR, Ayoub P (2006). Patient safety in outpatient surgery: the viewpoint of the healthcare providers. Ergonomics.

[38] Griffith CH III, Wilson JF, Desai NS, Rich EC (1999). Housestaff workload and procedure frequency in the neonatal intensive care unit. Crit Care Med.

[39] Baggs JG, Schmitt MH, Mushlin AI, Mitchell PH, Eldredge DH, Oakes D, Hutson AD (1999). Association between nurse-physician collaboration and patient outcomes in three intensive care units. Crit Care Med.

[40] Cavanagh SJ (1992). Job satisfaction of nursing staff working in hospitals. J Adv Nurs.

[41] Ouyang YQ, Zhou WB, Qu H (2015). The impact of psychological empowerment and organisational commitment on Chinese nurses’ job satisfaction. Contemp Nurse.

[42] Wynd CA (2003). Current factors contributing to professionalism in nursing. J Prof Nurs.

[43] Inoue KC, Gomes da Silva Versa GL, Misue Matsuda L (2014). Stress level among intensive care nurses in the municipality of Paraná (Brazil). Invest Educ Enferm.

[44] Vincent C, Taylor-Adams S, Stanhope N (1998). Framework for analysing risk and safety in clinical medicine. BMJ.

[45] Carayon P, Gurses AP (2008). Nursing workload and patient safety—a human factors engineering perspective. Patient Safety and Quality: An Evidence-Based Handbook for Nurses.

[46] Carayon P, Schoofs Hundt A, Karsh BT, Gurses AP, Alvarado CJ, Smith M, Flatley Brennan P (2006). Work system design for patient safety: the SEIPS model. BMJ Qual Saf.

[47] Clements A, Halton K, Graves N, Pettitt A, Morton A, Looke D, Whitby M (2008). Overcrowding and understaffing in modern health-care systems: key determinants in meticillin-resistant Staphylococcus aureus transmission. Lancet Infect Dis.

